# Zika Virus: An Emerging Global Health Threat

**DOI:** 10.3389/fcimb.2017.00486

**Published:** 2017-12-08

**Authors:** Rahul Mittal, Desiree Nguyen, Luca H. Debs, Amit P. Patel, George Liu, Vasanti M. Jhaveri, Sae-In S. Kay, Jeenu Mittal, Emmalee S. Bandstra, Ramzi T. Younis, Prem Chapagain, Dushyantha T. Jayaweera, Xue Zhong Liu

**Affiliations:** ^1^Department of Otolaryngology, University of Miami Miller School of Medicine, Miami, FL, United States; ^2^Department of Surgery, Division of Otorhinolaryngology, Dr. Kiran C. Patel College of Osteopathic Medicine, Nova Southeastern University, Fort Lauderdale, FL, United States; ^3^Division of Neonatology, Department of Pediatrics, University of Miami Miller School of Medicine, Miami, FL, United States; ^4^Department of Pediatrics, University of Miami Miller School of Medicine, Miami, FL, United States; ^5^Department of Physics and Biomolecular Sciences Institute, Florida International University, Miami, FL, United States; ^6^Department of Medicine, University of Miami Miller School of Medicine, Miami, FL, United States

**Keywords:** Zika virus, pathogenesis, animal models of Zika virus, Zika virus proteins

## Abstract

Zika virus (ZIKV) is an emerging healthcare threat. The presence of the mosquito *Aedes* species across South and Central America in combination with complementary climates have incited an epidemic of locally transmitted cases of ZIKV infection in Brazil. As one of the most significant current public health concerns in the Americas, ZIKV epidemic has been a cause of alarm due to its known and unknown complications. At this point, there has been a clear association between ZIKV infection and severe clinical manifestations in both adults and neonates, including but not limited to neurological deficits such as Guillain-Barré syndrome (GBS) and microcephaly, respectively. The gravity of the fetal anomalies linked to ZIKV vertical transmission from the mother has prompted a discussion on whether to include ZIKV as a formal member of the TORCH [Toxoplasma gondii, other, rubella virus, cytomegalovirus (CMV), and herpes] family of pathogens known to breach placental barriers and cause congenital disease in the fetus. The mechanisms of these complex phenotypes have yet to be fully described. As such, diagnostic tools are limited and no effective modalities are available to treat ZIKV. This article will review the recent advancements in understanding the pathogenesis of ZIKV infection as well as diagnostic tests available to detect the infection. Due to the increase in incidence of ZIKV infections, there is an immediate need to develop new diagnostic tools and novel preventive as well as therapeutic modalities based on understanding the molecular mechanisms underlying the disease.

## Introduction

**Figure 1:**
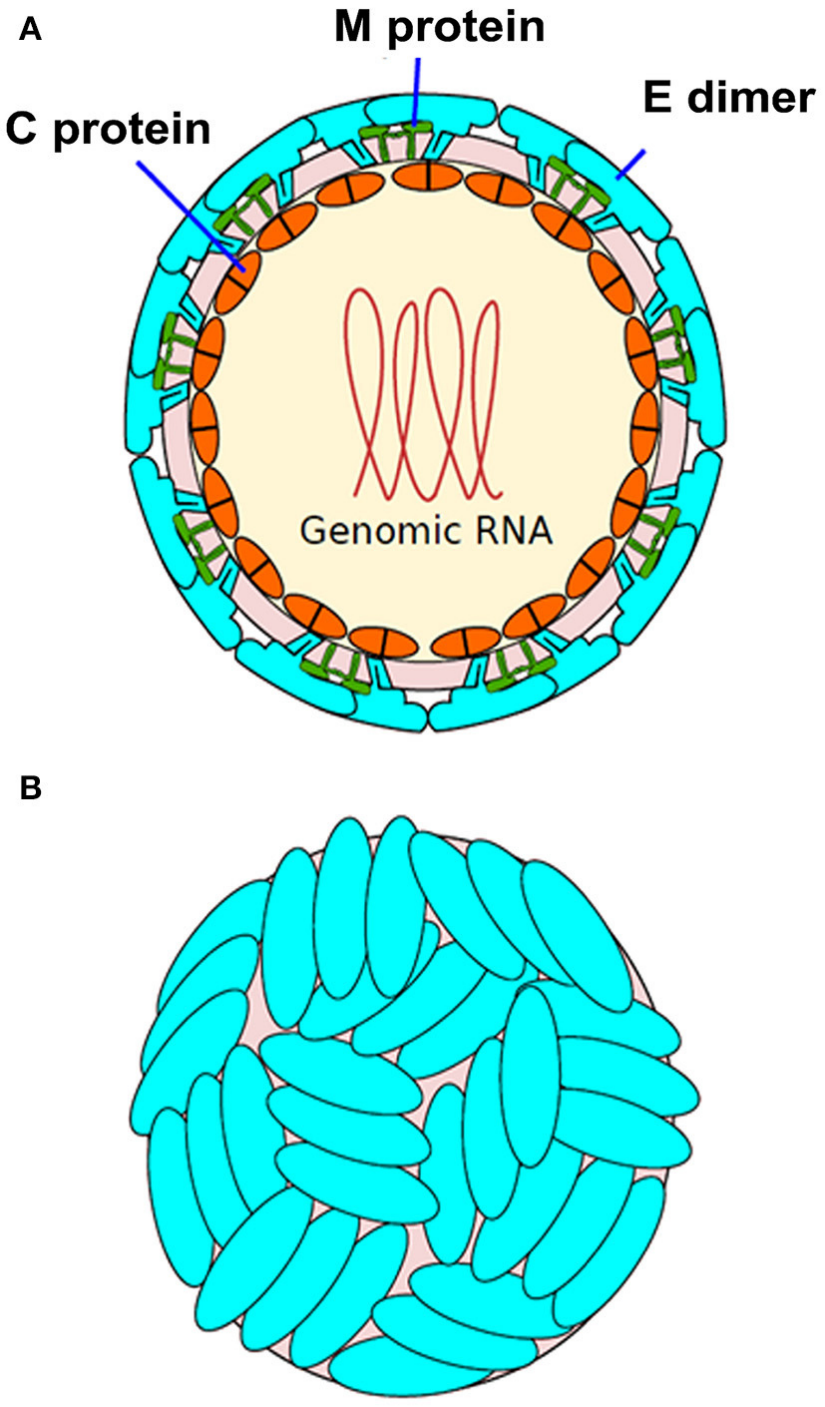
Structure of Zika virus (ZIKV). **(A)** The structure of ZIKV is comparable to many other flaviviruses. The nucleocapsid's diameter is ~25–30 nm. The envelope proteins E and M and other surface proteins are arranged in an icosahedral pattern. **(B)** Surface dimers depict T3-like organization (adapted from http://viralzone.expasy.org/all_by_species/6756.html).

**Figure 2:**
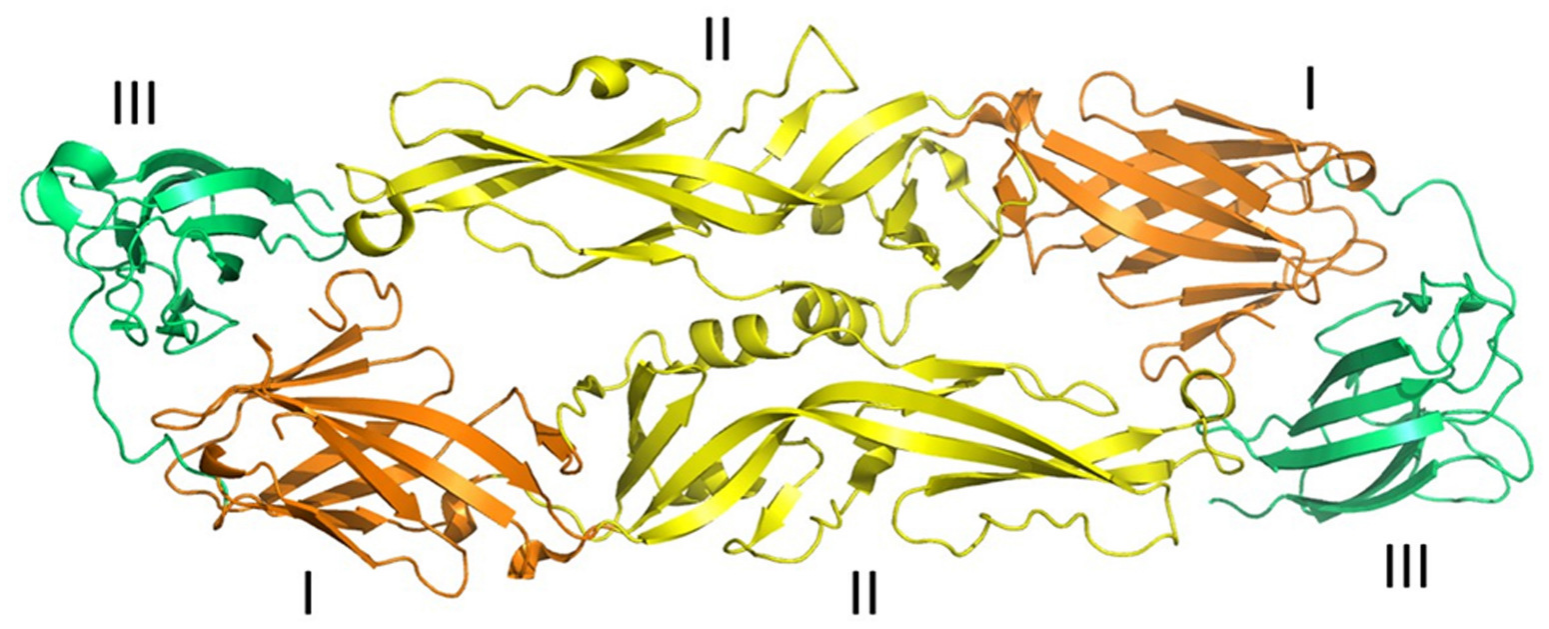
Three-dimensional structure of ZIKV envelope protein. Ribbon diagram of ZIKV Envelope Protein (ZIKV-E) dimer obtained from Protein data bank (PDB id: 5jhm). ZIKV-E has three distinct domains a central β-barrel (domain I; brown color), an elongated finger-like structure (domain II; yellow color), and a C-terminal immunoglobulin- like module (domain III; green color) (adapted from Dai et al., 2016). Protein structures were visualized and rendered with PyMOL 1.7.3 (The PyMOL Molecular Graphics System, Schrödinger, LLC).

Zika virus (ZIKV) belongs to the larger group of arboviruses (arthropod borne viruses). The *Aedes* genus of mosquitoes is the primary route by which ZIKV is transmitted (Chen and Tang, 2016; Musso and Gubler, 2016). Transmission of ZIKV has largely been observed through *Aedes aegypti* species. However, *Aedes albopictus* species (*Stegomyia albopicta*) have also been found to harbor ZIKV (Grard et al., 2014). The virus is a member of the Flaviviridae family and the genus *Flavivirus*. Compared to the other viruses from the same genus, only minor genomic divergence is noted (Weaver et al., 2016). ZIKV is a positive-sense RNA that is single stranded. Only a single polyprotein is encoded by its 10.7 kb genome. It is further cleaved into 10 proteins, three of which are structural (C, prM/M, E) and seven of which are non-structural (NS1, NS2A, NS2B, NS3, NS4A, NS4B, and NS5) (Figure 1; Faria et al., 2016). The crystal structure of ZIKV envelope is similar to other flaviviruses and possess three distinct domains: a central β-barrel-shaped domain I, an elongated finger-like domain II, and a C-terminal immunoglobulin-like domain III (Figure 2; Dai et al., 2016). Two main lineages have been identified so far from entire genome sequencing namely, the African and the Asian lineages. Further divided into two groups, the African lineage is composed of clusters Uganda and Nigeria. Most of the strains belonging to the African lineage were isolated from enzootic vectors, whereas the Asian lineage has been associated with the major human epidemics reported until now (Weaver et al., 2016). The new strains that have been recently isolated from Central and South America have shown a progressively more wide and rapid genetic divergence that can be attributed to their introduction into an immunologically naïve population (Zammarchi et al., 2016).

Before spreading to the Americas and the Pacific Island, ZIKV was contained through a sylvatic cycle. In Africa, where the sylvatic cycle still remains, the prevalence of enzootic transmission is high; the main reservoir is found in non-human primates while the vectors are primarily various species of forest mosquitoes (Diallo et al., 2014; Anderson et al., 2016). Cases of human infection in this setting are rare. However, the epidemics documented in French Polynesia, Micronesia, and the Americas are sustained by an urban cycle and therefore, are a much greater threat for transmission to humans (Duffy et al., 2009; Mallet et al., 2015; Zanluca et al., 2015).

Starting in February 2015 with Brazil, the Western Hemisphere has been colonized by ZIKV infection (de Oliveira et al., 2017a,b). As of September 2016, 46 countries and/or territories have reported autochthonous transmission (Deseda, 2017). ZIKV has spread on the American continents following a similar pattern as observed with Chikingunya and West Nile viruses. There were 41,181 confirmed Zika cases in the US according to the Centers for Disease Control and Prevention (CDC) in 2016 (https://www.cdc.gov/zika/reporting/2016-case-counts.html). This includes 5,102 cases from the mainland United States (of which 4,830 originated from returning travelers) and 36,079 cases in the American territories of Virgin Islands, Puerto Rico, and Samoa. In late July 2016, the first North American spread of Zika through mosquitoes was reported in Miami, Florida (McCarthy, 2016). The majority of the cases in the US states were not autochthonous. Related to the neurological disorders and microcephaly cases in Brazil (de Oliveira et al., 2017a,b), the World Health Organization (WHO) and the Pan American Health Organization have issued alerts about risks and prevention associated with ZIKV infection (Sikka et al., 2016; Vest, 2016).

## Epidemiological data on ZIKV

ZIKV was first detected in 1947 at the Yellow Fever Research Institute in Entebbe, Uganda, in a rhesus monkey blood. The following year it was detected again in the *Ae. Africanus* vector (Imperato, 2016; Mlacker et al., 2016). In Nigeria, the first human case was reported in 1954. It was discovered during a jaundice outbreak investigation. Later on, the presence of ZIKV in humans was confirmed as more cases throughout Africa and Asia arose (Zammarchi et al., 2016). It was not until 2007 in Micronesia, in the Yap islands, that the first major outbreak was reported affecting ~75% of the residents (Lanciotti et al., 2008; Duffy et al., 2009). Sporadic cases were subsequently reported in Cambodia, Indonesia, Thailand, East Malaysia, and the Philippines (Plourde and Bloch, 2016). A second large outbreak affected French Polynesia between October 2013 and April 2014, where about 11% of the population was estimated to be infected (Mallet et al., 2015). Subsequently, the virus spread to many other Pacific Islands (Plourde and Bloch, 2016). In early 2015, in the State of Rio Grande do Norte, ZIKV was reported for the first time in the Americas; as well as in a traveler returning to Italy from Bahia in the same period (Zammarchi et al., 2015; Zanluca et al., 2015). The indigenous cases of ZIKV infection were reported in October 2015 in Columbia followed by other South and Central American countries. Currently, the WHO states that transmission from continuous vector is present in 60 countries including most of Central and South America, South East Asia, Oceania, and the Pacific as well as in Africa, Cape Verde, and Guinea Bissau (Zammarchi et al., 2015).

Consistent among various models and representing the evolution of ZIKV, the rates of nucleotide substitutions per year are estimated to range between 0.98 × 10^−3^ and 1.06 × 10^−3^. As epidemics progress, their evolutionary rates tend to follow a progressively decreasing trend (Faria et al., 2016). It has been suggested that viral genetic features alone are not responsible for fetal abnormalities (Faria et al., 2016). However, a recent study demonstrated that single serine to asparagine substitution (S139N) in the viral polyprotein can lead to enhanced infectivity of human and mouse neural progenitor cells (NPCs) by ZIKV (Yuan et al., 2017). It was also suggested that this functional adaptation can lead to microcephaly in the mouse fetus, and higher mortality in neonatal mice. Further studies are warranted to decipher the contribution of genetic substitutions in viral genome to microcephaly and other ZIKV associated complications.

## Routes of transmission

While ZIKV transmission mainly occurs via the *Aedes* mosquitoes, precautions taken to avoid contact with infected insects are not enough. Indeed, there is a strong evidence that ZIKV can be transmitted in numerous ways, especially including oral, vaginal, and anal sexual contact. Guidance papers on recommended precautions were issued by the CDC and WHO based on available knowledge of ZIKV transmission. Abstinence for a minimum of 8 weeks and up to 6 months is advised in people returning from areas of known local transmission. The same considerations apply for couples planning a pregnancy (Petersen et al., 2016). When pregnant, unprotected sexual intercourse with a partner that was exposed to risks of infection should be avoided during the entire duration of gestation (Petersen et al., 2016; Zammarchi et al., 2016).

Furthermore, transmission of the virus through various substances of human origin such as blood product is very likely. Although the transmission potential has not been demonstrated yet, the virus has been found in saliva, breast milk, semen, and urine (Musso et al., 2015; Barzon et al., 2016; Dupont-Rouzeyrol et al., 2016; Rozé et al., 2016). A small number of infections were reported in the context of laboratory accidents, and one in the event of a bite from a monkey (Filipe et al., 1973; Leung et al., 2015). Healthcare professionals and authorities involved with the distribution of donor-derived human substances must maintain awareness of the possibility of ZIKV transmission thorough human substances, and should implement precautions accordingly. The European CDC has recently released a document summarizing information on how donor-derived human substances were managed to prevent the distribution of other Arboviruses during previous outbreaks. This information is highly relevant for the implementation of ensuring ubiquitous prevention of transmission through this unique route (Zammarchi et al., 2016).

## Clinical presentation of ZIKV infection

Of all the known cases of ZIKV infections, 80% are asymptomatic (Table 1). The symptoms and signs of patients affected by ZIKV are similar to Dengue virus and other viral diseases. The patient may present with fever, body aches, joint pains, fatigue, malaise, and conjunctivitis (Table 1). As with other viral illnesses, maculopapular rash can also occur. Total duration of illness can last up to 5–7 days (Jamil et al., 2016). Symptoms are observed in 50% of individuals after 5.8 days and in 95% after 11.2 days following exposure (Lessler et al., 2016). However, most infections take a benign course and do not cause any complications. In most instances, symptoms are resolved within a period of 2 weeks. Maculopapular rashes of duration from 4 to 5 days are the most common reported manifestation of the disease (90% of cases). While often itchy and evolving in a centrifugal manner, they can sometimes be preceded by symptoms of fatigue and low fever (Duffy et al., 2009; Mallet et al., 2015; Plourde and Bloch, 2016; Waddell and Greig, 2016). Peripheral edema of the joints is often observed and cause symptoms resembling those of arthralgia which commonly subside within a week. Associated with as many as half of the cases, conjunctival infection without pus can occur in the early time period (Duffy et al., 2009; Mallet et al., 2015). The cases of lymphadenopathy were also found in infected pregnant Brazilian women to be as frequent as 40%. Myalgia and headaches are also highly common (Mallet et al., 2015). The congenital manifestations of ZIKV include microcephaly, ventriculomegaly, intracranial calcifications, abnormalities of the corpus callosum, retinal lesions, craniofacial disorder, hearing loss, and dysphagia (Table 2).

**Table 1:** Clinical manifestations of ZIKV infection in children and adults.

**Symptom**	**Estimated incidence**	**Demographics**	**References**
Asymptomatic	Majority	Adults	Brasil et al., 2016; Gallian et al., 2017; Joob and Wiwanitkit, 2017; Moghadas et al., 2017
Maculopapular pruritic rash	Most symptomatic individuals	Adults	Edupuganti et al., 2017; Ioannou et al., 2017; Yoon et al., 2017; Cosano-Quero et al., in press
		Children	
Low-grade fever	Most symptomatic individuals	Adults	Brasil et al., 2016; Petersen et al., 2016; Grossi-Soyster and LaBeaud, 2017
		Children	
Arthralgia/Arthritis of small joints	Most symptomatic individuals	Adults	Brasil et al., 2016; Petersen et al., 2016; Grossi-Soyster and LaBeaud, 2017; Ioannou et al., 2017; Tyler and Roos, 2017
		Children	
Non-purulent conjunctiviits	Most symptomatic individuals	Adults	de Paula Freitas et al., 2017b; Marquezan et al., in press
		Children	
Myalgia	Common	Adults	Guerbois et al., 2016; Garza-González et al., 2017; He et al., 2017
		Children	
Retro-orbital pain	Common	Adults	Bachiller-Luque et al., 2016
		Children	
Fatique	Common	Adults	Sokal et al., 2016
		Children	
Headache	Common	Adults	Brasil et al., 2016; Petersen et al., 2016; Grossi-Soyster and LaBeaud, 2017
		Children	
Vomitting	Uncommon	Adults	Pougnet et al., 2016; Murray, 2017
		Children	
Nausea	Uncommon	Adults	Brasil et al., 2016; Petersen et al., 2016; Grossi-Soyster and LaBeaud, 2017
		Children	
Abdominal pain	Uncommon	Adults	Brasil et al., 2016; Petersen et al., 2016; Grossi-Soyster and LaBeaud, 2017
		Children	
Diarrhea	Uncommon	Adults	Bachiller-Luque et al., 2016; Murray, 2017
		Children	
Mucous membrane ulcers	Uncommon	Adults	Brasil et al., 2016; Petersen et al., 2016; Grossi-Soyster and LaBeaud, 2017
		Children	
Facial edema	Rare	Adults	Zammarchi et al., 2015; Grossi-Soyster and LaBeaud, 2017
		Children	
Palatal petechiae	Rare	Adults	Derrington et al., 2016; Grossi-Soyster and LaBeaud, 2017
		Children	
Uveitis	Rare	Adults	Furtado et al., 2016; Grossi-Soyster and LaBeaud, 2017
		Children	
Desquamation of palms	Rare	Adults	da Cunha et al., 2016; Grossi-Soyster and LaBeaud, 2017
		Children	
Guillain-Barre syndrome (GBS)	Proposed	Adults	Arias et al., 2017; da Silva et al., 2017; Nascimento and da Silva, 2017; Ugarte et al., 2017
		Children	
Severe pruritis	Rare	Adults	Farahnik et al., 2016
Gastrointestinal or respiratory complication	Rare	Adults	Savino et al., 2017

**Table 2:** A summary of Congenital ZIKV syndrome (CZS) manifestations.

**Symptom**	**Estimated incidence**	**Demographics**	**References**
Microcephaly	Unknown	Neonates	Ventura et al., 2016a; Moi et al., 2017; Sousa et al., 2017
Craniofacial disproportion	Unknown	Neonates	Costello et al., 2016; Ozkurt and Tanriverdi, 2017
Redundant scalp	Unknown	Neonates	Costello et al., 2016; Calotta et al., 2017
Closed fontanel	Unknown	Neonates	Nielsen-Saines et al., 2012; Melo et al., 2016
Hearing loss	Unknown	Neonates	Leal et al., 2016; Mittal et al., 2017; Wiwanitkit, 2017
Retinal, macular and optic nerve defects, cortical/cerebral visual impairment	Unknown	Neonates	de Paula Freitas et al., 2016; Ventura et al., 2016a,b, 2017; Yepez et al., 2017
Club foot	Unknown	Neonates	Rasmussen et al., 2016
Irritability	Common	Neonates	Nielsen-Saines et al., 2012; Costello et al., 2016; de Paula Freitas et al., 2016; Moshfeghi et al., 2016; Aragao et al., 2017; Moore et al., 2017
Hypertonia	Common	Neonates	Nielsen-Saines et al., 2012; Costello et al., 2016; de Paula Freitas et al., 2016; Moshfeghi et al., 2016; Aragao et al., 2017; Moore et al., 2017
Hyperreflexia	Common	Neonates	Miranda-Filho Dde et al., 2016
Feeding difficulties	Common	Neonates	Nielsen-Saines et al., 2012; Costello et al., 2016; de Paula Freitas et al., 2016; Moshfeghi et al., 2016; Aragao et al., 2017; Moore et al., 2017
Dysphagia	Common	Neonates	van der Linden et al., 2016; Leal et al., 2017
Seizures	Rare	Neonates	Asadi-Pooya, 2016; Rozé et al., 2016; Carvalho et al., 2017
Intrauterine growth retardation	Common	Neonates	Brasil et al., 2016; Mayor, 2016; Sarno et al., 2016

## Complications

**Figure 3:**
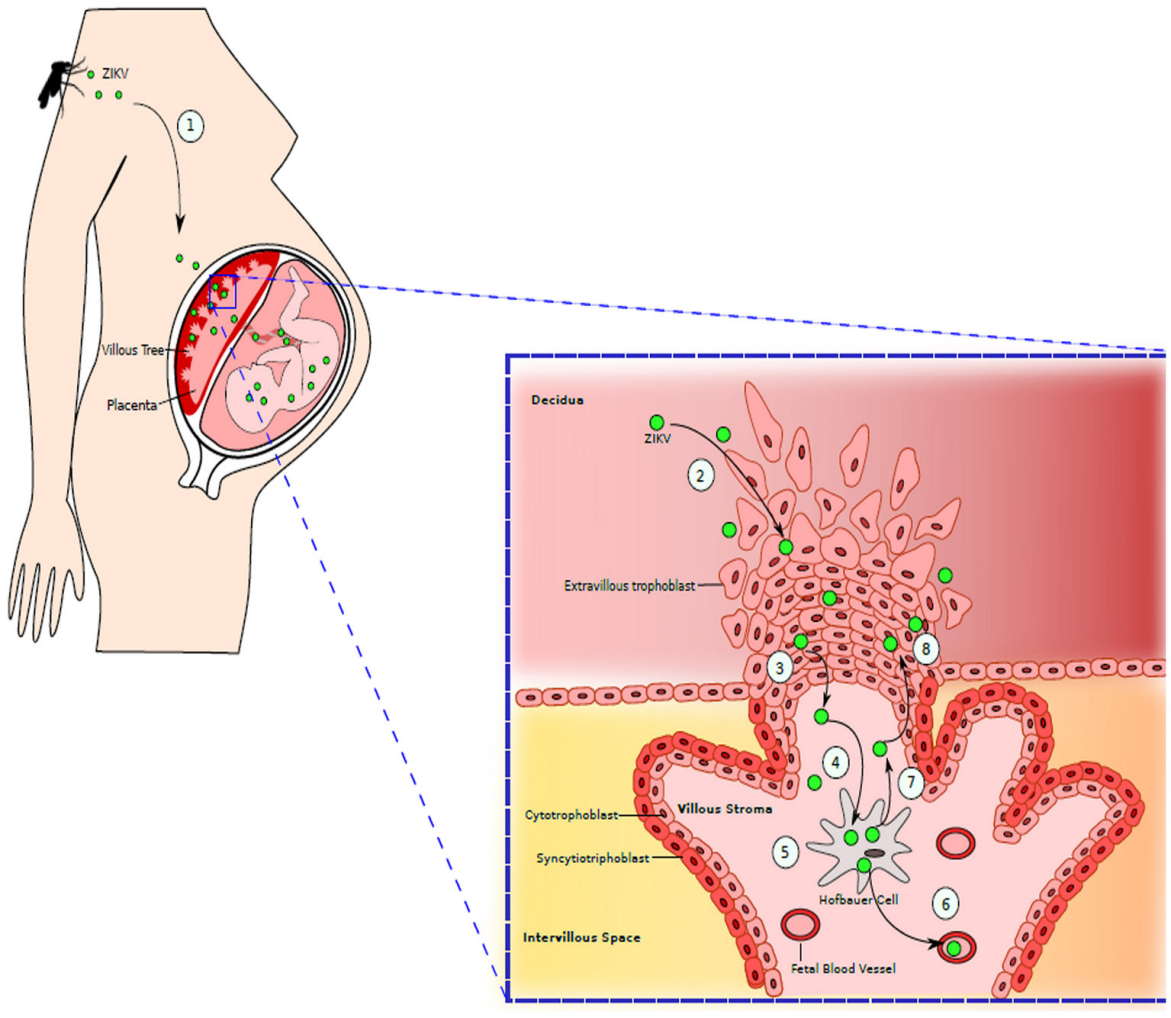
Maternal-Fetal Zika Virus Transmission: (1) Mother is bitten by the mosquito infected with ZIKV. ZIKV is spread throughout the maternal vasculature. (2) ZIKV gains access to the placenta by infecting extravillous trophoblasts residing in decidua. (3) ZIKV moves further into the stroma of villous tree of placenta. (4) ZIKV then infects the placental macrophages, Hofbauer cells. (5) ZIKV replicates inside Hofbauer cells. (6) When new generation of ZIKV is released from the macrophages, it can further migrate to fetal blood vessels, infecting the fetus. (7) ZIKV released from Hofbauer cell can also migrate back to extravillous trophoblast. (8) Newly released ZIKV further infects and damages the trophoblast layer, granting an easy access to placenta by ZIKV. Adapted from Coyne and Lazear (2016).

A growing body of evidence indicates that ZIKV is transmissible vertically, during pregnancy through the placenta in the perinatal period (Figure 3; Besnard et al., 2014; Calvet et al., 2016). Indeed, in newborns from women who became infected while in gestation, viral RNA was detected via Polymerase chain reaction (PCR) in the central nervous system, fetal tissues, and amniotic fluid (Mlakar et al., 2016). The most serious complication of ZIKV infection in pregnant women, which has become an alarming signal for the global health authorities, is its association with microcephaly and other severe neurological complications in the developing fetus. Pregnant mothers residing in or traveling to endemic areas may present with microcephaly or low-birth-weight babies due to infection by ZIKV (Blázquez and Saiz, 2016; Panchaud et al., 2016; Hajra et al., 2017). This transmission has been proven after isolating virus from fetal and placental tissues as well as from amniotic fluid and cerebrospinal fluid of infected newborns (Calvet et al., 2016; Sarno et al., 2016; van der Eijk et al., 2016; Reagan-Steiner et al., 2017). ZIKV has been demonstrated to infect the primary progenitor cells of the neurological system, thus preventing their growth and explaining the most likely reason for microcephaly. In Brazil alone, 4,000 cases of microcephaly have been suspected to be due to the virus (Werner et al., 2016). In America, the presence of ZIKV has been confirmed by the CDC in nine pregnant females who visited virus-infected areas during pregnancy (Meaney-Delman et al., 2016). While one of them gave birth to a newborn with microcephaly, another two had miscarriages and rest two underwent medical termination of pregnancy after confirmation of fetal microcephaly. Infection of the fetus can have many impacts on pregnancy and are not limited to microcephaly. Adverse outcomes like placental insufficiency, early abortions, and intrauterine growth retardation have also been reported (Jamil et al., 2016; Hajra et al., 2017). A newly emerging hypothesis regarding the effects of ZIKV on placental signaling during embryogenesis could explain the changes seen in infants born to infected mothers. This hypothesis highlights the dangers of infection during the first trimester of pregnancy, when most of the observable damages seem to occur (Adibi et al., 2016).

Several case reports discuss the potential transmission of the disease from both symptomatic and asymptomatic males to their partners via sexual intercourse (Deckard et al., 2016; Fréour et al., 2016; Venturi et al., 2016). Using the virus isolation technique and PCR, the virus could be detected in sperm for up to 24 and 188 days, respectively (Nicastri et al., 2016). In America, transmission from a female carrier to a male partner was reported for the first time. This comes with no surprise as the virus can be readily detected in vaginal and cervical mucosa only a few days after onset of symptoms (Davidson et al., 2016; Prisant et al., 2016).

The current outbreak of ZIKV has rendered the medical community strongly concerned by the increased incidence of Guillain-Barré syndrome (GBS). ZIKV is considered an etiology for GBS, an acute medical condition that can lead to lifetime disability or death due to respiratory muscle involvement. In 2013–2014, an outbreak in French Polynesia was the first instance during which the correlation between ZIKV infection and GBS was made. Over a period of 4 months, 42 cases were registered. Before 2013, the yearly average was only of 5 infections (Barzon et al., 2016). According to the data gathered following the French Polynesia epidemic, 2.4 individuals among an infected population of 10,000 would be expected to develop GBS (Waddell and Greig, 2016). The incubation period for GBS after the appearance of ZIKV symptoms was ranging between 2 and 23 days with a median of 6 (Zammarchi et al., 2016).

At this stage, it is unclear if ZIKV alone is the causative agent GBS, or if conjunction with other viruses is required (Jamil et al., 2016). Severe or life-threatening events are not reported frequently and are exceptionally rare; they are solely limited to patients with comorbidities. Amongst such events there are two reported deaths in patients, one suffering from sickle cell disease, and another one suffering from various chronic illnesses on long term corticosteroid treatment. There are also four cases of neuro-invasive disease (including two cases of encephalopathy, one case of meningoencephalitis in a senior patient, and one case of myelitis in a very young patient) and two fatal cases in patients with severe comorbidities (a child with sickle cell disease and a man with alcoholism, systemic lupus erythematosus, and rheumatoid arthritis in chronic treatment with corticosteroids; Arzuza-Ortega et al., 2016; Carteaux et al., 2016; Mécharles et al., 2016; Rozé et al., 2016). Further studies are warranted to confirm the role of ZIKV infection in causing GBS and other neurological manifestations including microcephaly.

## Pathogenesis of ZIKV infections

Unfortunately, very little is understood about the pathophysiology of ZIKV. Entry of the ZIKV through human skin has been studied in dermal fibroblasts, immature dendritic cells, and epidermal keratinocytes, where it was found that entry and adhesion factors, such as Tyro-3, DC-SIGN, AXL, and even TIM-1, were responsible for ZIKV penetration (Hamel et al., 2015). Furthermore, the study demonstrated a role for the phosphatidylserine receptor AXL, where its presence allowed ZIKV entry and cellular autophagy into permissive cells (Hamel et al., 2015). ZIKV has also been observed to antagonize type I interferon signaling in human cells that plays a crucial role in host innate antiviral immunity (Grant et al., 2016; Kumar et al., 2016). The underlying molecular mechanism involves binding of viral protein NS5 to STAT2 followed by its degradation. STAT2 is a signal transducer and activator of transcription in mammals leading to the enhanced expression of hundreds of IFN-stimulated genes (ISGs; MacMicking, 2012). In this manner, ZIKV employs pathogenic mechanisms similar to Dengue virus (Grant et al., 2016). However, unlike Dengue virus, ZIKV does not employ the E3 ubiquitin ligase UBR4 to induce STAT2 degradation (Grant et al., 2016).

ZIKV replication primarily begins in the cytoplasm of mammalian cells, when protein E binds to the host cell membrane receptors and host-mediated endocytosis of the virus is initiated (Perera et al., 2008). Replication of ZIKV will activate the antiviral innate immune response in the affected cells, which leads to type I interferon assembly (Hamel et al., 2015). This replication is further enhanced by autophagosome formation itself, as well as the expression of several antigen clusters that enhance antiviral activity, such as MDA-5, RIG-1, and TLR3, is induced due to early detection of pathogen-associated molecular patterns within skin fibroblasts (Hamel et al., 2015). On the other hand, a study discovered that early ZIKV infection causes cells to produce interferon beta resulting in apoptosis of potential ZIKV host cells (Frumence et al., 2016). This could be one mechanism to further exploit in the quest of developing novel treatment modalities for ZIKV infection.

Due to the association of ZIKV infection with serious clinical manifestations, especially damaging neurological involvement, there is an urgency to grasp more fully the consequences of ZIKV infections within these systems. AXL mentioned earlier not only has a contributing role in viral entry within skin cells, but it also is located in large quantities in human radial glial cells, cortical astrocytes, blood microcapillaries, and microglia *in vitro* (Nowakowski et al., 2016). ZIKV infection augments the number of AXL in fetal mouse brain tissues suggesting a clear association between ZIKV infection and the virus mode of access to a developing fetal brain (Li et al., 2016). A correlation between ZIKV infection and neural cell proliferation and induction of apoptosis has also been demonstrated (Cugola et al., 2016). While evaluating the brains of mice pups on Swiss Jim Lambert (SJL) background whose mothers were infected with ZIKV, genes responsible for autophagy and apoptosis, such as *Bmf*, *Irgm1, Bcl2, Htt, Casp6*, and *Abl1*, were found to be upregulated (Cugola et al., 2016). *In vivo* studies involving the infection of human neural progenitor cells and *in vitro* studies involving cells in the intermediate zone and cortical plate of embryonic mouse brains found that genes associated with apoptosis were upregulated, namely caspase-3/7, as determined by RNA analysis (Garcez et al., 2016; Li et al., 2016; Tang et al., 2016). It can also be speculated that ZIKV targets post-mitotic neurons as well, since these are located on the cortical plate (Li et al., 2016). In addition, ZIKV involvement in the regulation of neurogenesis and apoptotic genes is further established by activating toll-like receptor 3 (TLR3) (Dang et al., 2016). The role of TLR3 indirectly causes apoptosis by inhibiting Sonic Hedgehog (SHH) and Ras-ERK signal transduction pathways found in neural progenitor cells (Shiose et al., 2011; Yaddanapudi et al., 2011). Upregulation of *tlr3* gene expression was observed in ZIKV-infected brains of fetal mice (Li et al., 2016). In addition, upregulation of cytokines namely, interleukin (IL)-1β, IL-2, IL-4, IL-6, IL-9, IL-10, IL-13, IL-17, as well as interferon-γ-induced protein 10 (IP-10), regulated on activation, normal T cell expressed and secreted (RANTES), macrophage inflammatory protein 1 alpha (MIP-1α) and vascular endothelial growth factor (VEGF) have been observed in serum of patients during acute phase of ZIKV infection compared to normal uninfected individuals (Tappe et al., 2016). The cytokines return to baseline during recovery (reconvalescent) phase. These findings suggest that activation of immune responses can play a crucial role in recovery from ZIKV infection. However, further studies are warranted to confirm these findings.

The discussion about how ZIKV affects neural tissue is imperative to further understand one of the most devastating birth defects in newborns—microcephaly. It is challenging to assess how the phenotype is established due to the multifactorial and complicated components of microcephaly. A study reported some interesting findings about ZIKV proteins that may warrant additional investigation (Lucchese and Kanduc, 2016). The altered penta- and hexapeptides of the ZIKV polyprotein were utilized to find homology with human-derived proteins. They found several regions of homology that were correlated with microcephaly as well as calcifications within the brain. Similarly, ZIKV peptide homology occurrences in human proteins were also associated with Guillain-Barré-like syndromes, displaying an elevated level of peptide sharing. Perhaps one of the more significant findings from this study demonstrated that the shared peptides had immunological potential, such that ZIKV infection could trigger an immune reaction and the components of these reactions could go on to cross-react with brain-specific proteins. It is believed that these immune components play a role in the sequelae of ZIKV-triggered neuropathological events. While this study warrants further investigations to determine if the proposed hypotheses are correct, it provides novel clues about the pathogenesis of ZIKV-induced neurogenic disease. The studies have demonstrated the downregulation of a number of genes linked to microcephaly, including, *microcephalin, Aspm, Casc5, Cenpf, Mcph1, Rbbp8, Stil*, and *Tbr2* (Li et al., 2016; Wu et al., 2016). Further, studies are warranted to determine the molecular mechanisms that lead to microcephaly during ZIKV infection.

In addition to the microcephaly, ocular manifestations of ZIKV infection have become a source of concern (de Paula Freitas et al., 2016, 2017a; Yepez et al., 2017). The first case of ocular lesions associated with presumed ZIKV infection were reported in three children with microcephaly born after the ZIKV outbreak in Brazil. There was focal pigment mottling observable in the macular region in all three children and a well-defined macular chorioretinal atrophy was also found in one infant (Ventura et al., 2016a). In later studies, the same group observed ocular complications in 10 infants who had been diagnosed clinically with ZIKV-related microcephaly (Ventura et al., 2016b). The macular pigment mottling and/or chorioretinal atrophy was observed in 75% of cases. There were optic nerve abnormalities such as optic disc hypoplasia, pallor, and/or increased cup-to-disc ratio in 45% of cases. In another study, ZIKV babies who develop macular defects were found to have serious involvement of the neurosensory retina and choroid when observed with optical coherence tomography (OCT) (Ventura et al., 2016c). The discontinuation of the ellipsoid zone and hyperreflectivity underlying the retinal pigment epithelium, retinal as well as choroidal thinning, and colobomatous-like excavation involving the neurosensory retina as well as retinal pigment epithelium was observed using OCT. Studies have also associated glaucoma with congenital ZIKV syndrome (CZS) (de Paula Freitas et al., 2017a; Yepez et al., 2017). A recent study demonstrated histopathological abnormalities within the eyes of ZIKV infected fetuses (Fernandez et al., 2017). The eyes of these ZIKV infected fetuses showed poor differentiation and maturation of the pupillary membrane, photoreceptor layer, and nuclear layers of the neural retina as well as thinning of the retinal pigment epithelium and choroid. The pathogenesis of these findings remains unclear, but the presence of ZIKV in each of these layers seems to suggest that ZIKV is responsible for these ocular deformities (Fernandez et al., 2017). Future investigations will shed light on the molecular mechanisms underlying ocular complications during ZIKV infection.

## ZIKV and the onset of inflammatory events in the placenta

ZIKV infection in pregnant women has caused major distress and propagated a need to understand the link between ZIKV from maternal blood and the trigger of catastrophic fetal abnormality onset. The placenta is both a physical and immunological barrier, separating the maternal and fetal compartments (Burton and Jauniaux, 2015; Roberts et al., 2016). The cells with direct contact to the maternal blood are called syncytiotrophoblasts and these are considered the key defense component for the fetus. Despite this protection, one can deduct that ZIKV must have the capability to access the fetal compartment to trigger the fetal abnormalities. Amniotic fluid as well as placental tissue has been evaluated over different gestational times and it has been determined that ZIKV RNA can be present at any time over the course of the pregnancy (Calvet et al., 2016; Martines et al., 2016; Noronha et al., 2016; Sarno et al., 2016). Regardless, there appears to be a relationship with ZIKV-related birth defects and gestational age, as illustrated by the fact that pregnant women infected with ZIKV during the third trimester did not have babies with birth defects while babies infected in earlier gestational ages were born with severe abnormalities (Pacheco et al., 2016). While this association exists, further studies are needed to elucidate the underlying mechanism.

ZIKV must be able to traverse through many cell layers and tissue barriers in order to access the embryo during the first trimester of pregnancy. These cell layers include syncytiotrophoblasts, cytotrophoblasts, macrophages, and finally the fetal endothelial layer (Roberts et al., 2016). It is possible that, through transplacental passage or diffusion, ZIKV could pass into the amniotic and yolk sacs during embryogenesis, which would allow the virus to gain access to the fetal brain. Although, placental cells should, in theory, be protected from ZIKV infection if they exhibit a constitutive IFN-γ response, the possibility of a transplacental passage would not be excluded by this response (Bayer et al., 2016). ZIKV RNA was observed in different trophoblast cells of mouse placentas and under histological evaluation, the placentas were found to be smaller in size and the trophoblasts apoptotic (Miner et al., 2016). These histological observations of a fragmented placental barrier could be a point of entry for ZIKV. It remains unclear if the mechanisms that prevent infection by ZIKV are functional in early pregnancy. In fact, A recent *in vitro* study showed that primary human placental macrophages and cytotrophoblasts were allow for the propagation of ZIKV infection (Bayer et al., 2016; Quicke et al., 2016). Further *ex vivo* findings by Tabata et al. demonstrate that ZIKV can be detected in chorionic villi (Tabata et al., 2016). Researchers have previously reported ZIKV is unable to infect the more differentiated syncytiotrophoblasts (Bayer et al., 2016). Syncytiotrophoblasts were found to constitutively make type III interferons, especially IFN-γ1, which protect the cells against ZIKV *in vitro* (Bayer et al., 2016). Non-placental cells are also protected from the infection by this action of antiviral type II interferons (Bayer et al., 2016). It is possible that ZIKV may avoid type I and type III IFN-antiviral signaling demonstrated by the villous syncytiotrophoblasts and placental macrophages during late pregnancy. If this is true, this circumvention would allow for penetration into the fetal compartment (Bayer et al., 2016; Quicke et al., 2016). The evidence presented in these studies demonstrates that it is likely that ZIKV uses alternative approaches to navigate the placenta, most likely via maternal blood, rather than direct infection of the placenta.

Other routes of fetal ZIKV infection during pregnancy could include leakage through the trophoblastic plugs or movement of ZIKV into the amniotic and yolk sacs of the developing fetus, as these are common transplacental routes of infection (Adibi et al., 2016). Prior studies have confirmed the presence of ZIKV in the semen, which means the virus has early access to the embryo. Combined with the evidence for sexual transmission of ZIKV, this could possibly be a route for infection of the embryo, but it is unlikely that this is the major route (Musso et al., 2015).

It was demonstrated in previous studies that fetal brain and tissue may exhibit morphological changes secondary to inflammation if the placenta is infected with ZIKV. These changes were demonstrated even when the affected disuse demonstrated no infection (Mor, 2016). Inflammatory processes in the placenta may lead to altered expression and regulation of neuropeptides and growth factors. Such an effect would result in microcephaly if the involved factors and peptides played a role in normal brain development (Mor, 2016). Signals induced within the placenta affect the response of the maternal immune system to antigenic signals. The resultant inflammation may have systemic effects on the morphology of the fetus. In order to prevent mortality of the mother and injury to the fetus, approaches for treatment must address immune response within the mother, the fetus, and the placenta. Failure to address all three will result in inadequate prevention of consequences.

## Animal models to study ZIKV

To understand the pathogenesis of disease, availability of an animal model that closely mimics human clinical conditions is of utmost importance. A variety of experimental animal models have been developed to understand the pathogenesis of ZIKV infection (Table 3; Cugola et al., 2016; Musso and Gubler, 2016).

**Table 3:** A summary of animal models established to understand the pathogenesis of ZIKV.

**Animal**	**Animal model**	**ZIKV strain**	**Experiment goal**	**Measured change from control**	**Evidence of ZIKV infection**	**References**
Mouse	C57Bl/6	ZIKV SZ01	Observe birth defects in newborns from females infected intraperitoneally with ZIKV	Microcephaly like development Reduced proliferation of cortex founder cells	N/A	Wu et al., 2016
Mouse	Pregnant C57BL/6 variant live animal	ZIKV^BR^	Observe birth defects in newborns from females infected through footpads during pregnancy	No physical changes were observed CT scans showed normal Skull/body volumes	qPCR assay was negative	Cugola et al., 2016
Mouse	Pregnant SJL variant live animal	ZIKV^BR^	Observe birth defects in newborns from females infected through footpads during pregnancy	Whole body growth delay Cortical malformation Reduced brain cell number Reduced cortical layer thickness Dysregulation of apoptosis and autophagy-related genes	qPCR assay was positive for ZIKV RNA in multiple tissues including brain	Cugola et al., 2016
Mouse	A129 and AG129	ZIKV^BR^	Effect of ZIKV inoculation on 3 week old and adult A129/AG129 mice	For mice under 3 weeks old, ZIKV infection was found to be deadly within 7 days with pathology to brain and muscle 11 week old mice displayed viremia, weight loss, and illness, but recovered starting at 8 days after the infection	Plaque assay found virus in visceral tissues and brain	Aliota et al., 2016; Aman and Kashanchi, 2016; Rossi et al., 2016; Dowall et al., 2017
Mouse	*Ifnar1^−/−^* female mating with WT male	ZIKV^BR^	To observe birth defects from ZIKV inoculation of *Ifnar^+/−^* fetus	Infection led to fetal demise and low IUGR scores	RT-PCR and plaque assay found virus in placenta and fetal brain	Miner et al., 2016
Mouse	Pregnant WT female treated with anti-ifnar monoclonal antibody	ZIKV^BR^	Observe birth defects in newborns from females infected during pregnancy	No defects noted	RT-PCR and plaque assay found no evidence of infection	Miner et al., 2016
Chicken embryos	WT chicken embryos	ZIKV MEX1-44	Observe effects of infection on chicken embryo development	Lethality at high doses (>20 viral particles) Microcephaly-like presentation at low doses (2–20 viral particles)	Plaque assays demonstrated dramatic increases in viral load across both dose levels	Goodfellow et al., 2016
Chimpanzee	Brain organoids developed from pluripotent stem cells	ZIKV^BR^	Observe changes in the number of TBR 1- or CTIP2 positive cells	No reduction in cell percentages	No evidence of ZIKV replication	Cugola et al., 2016
Chimpanzee	Brain organoids developed from pluripotent stem cells	ZIKV^AF^	Observe changes in the number of TBR 1- or CTIP2 positive cells	Slight reduction in TBR 1- and CTP2 cell percentages	Evidence of ZIKV infection	Cugola et al., 2016
Rhesus Macaque	Non-pregnant WT	ZIKV strain H/PF/2013	Observe infection in healthy adult Rhesus Macaques	Increase in natural killer cells, CD8+ cells, CD4+ cells, and plasmablasts Neutralizing antibodies formed in all subjects at 21 days Rechallenge did not elicit infection	ZIKV RNA was detected in Urine, saliva, and CSF at 1 day post infection. At 21 days post infection, no ZIKV RNA was found	Dudley et al., 2016
Rhesus Macaque	Pregnant WT	ZIKV strain H/PF/2013	Observe effects of infection on pregnant females and fetus	Fetal IUGR scores are at the low end of normal for both fetuses	Infection was found until 57 days after infection in pregnant females. Amniocentesshows no ZIKV RNA in fetuses at 36 and 43 days post infection	Dudley et al., 2016
Dunkin-Hartley guinea pigs	Adult animals	A sequence-verified ZIKV strain, PRVABC59 (PR 2015)	Observe infection in healthy adult Guinea Pigs	Clinical signs of infection characterized by fever, lethargy, hunched back, ruffled fur, and decrease in mobility A dramatic increase in protein levels of multiple cytokines, chemokines and growth factors in the serum	ZIKV was detected in the whole blood and serum using qRT-PCR and plaque assay Anti-ZIKV neutralizing antibody was detected in the infected animals using PRNT	Kumar et al., 2017

Mouse models have been one of the most widely studied animal models in the pathogenesis of ZIKV. Though there are some issues with the homology of mouse models to human pathogenesis, these models have been well accepted because of numerous similarities in genetics and development between mice and humans. A primary concern of the recent ZIKV outbreak is concomitant rise in cases of neurodevelopment issues in newborns from areas with Zika outbreaks (Araujo et al., 2016). Studies in mouse models have shed some light on the pathogenesis of neurodevelopment issues involved with ZIKV (Al-Qahtani et al., 2016; Demir and Kilic, 2016; Fajardo et al., 2016; Musso and Gubler, 2016).

It has been demonstrated that the primary barrier to vertical transmission of ZIKV in mice is the placenta (Wu et al., 2016). Pregnant C57BL6 mouse models did not display vertical transmission when the mother was infected intravenously with Brazilian ZIKV strain (Cugola et al., 2016). However, the intraperitoneal injection of an Asian ZIKV strain SZ01 into pregnant C57BL6 mice resulted in marked decrease in proliferation of key cortical neural progenitors including Ki67 positive cells in the ventricular/subventricular zone and intermediate zone, BLBP positive cells, and Sox2 cells. Decreased levels of RNA of a variety of genes involved in cell cycle progression and microcephaly including *Microcephalin, CDK5RAP2, CASC5, ASPM, CENPJ, STIL, CEP135*, and *CDK6* was also observed (Wu et al., 2016). Morphologically, the outer perimeters of the cortex were found to be shorter, and the cavity of lateral ventricles as well as the ventricular surfaces were reduced. These findings clearly demonstrate the supposition that ZIKV vertical transmission is dependent on placental permeability to the virus (Lazear et al., 2016; Wu et al., 2016).

Other studies thus far have found that the transmission of ZIKV from mother to fetus in animal models is dependent on the genetic background of the mother and the fetus. C57BL6 mice have shown robust immunity to vertical transmission, but SJL and *Ifnar1*^−/−^ mice strains have demonstrated vertical transmission to varying degrees (Cugola et al., 2016; Miner et al., 2016). SJL mice produced viable pups, but the pups were seen to display whole body growth delay, cortical malformations, decreased numbers of brain cells, decreased cortical thickness, and dysregulation of apoptosis and autophagy related genes (Cugola et al., 2016). On the other hand, when WT mice were crossed with *Ifnar1*^−/−^ mothers, ZIKV infection was found to result in fetal demise (Miner et al., 2016). Interestingly, pups from WT mice injected with anti-*Ifnar* monoclonal antibody 1 day prior to infection demonstrated no defects as well as no evidence of infection in RT-PCR and plaque assays (Miner et al., 2016). The differential presentations of vertical transmissions, combined with the variable presentations of ZIKV infection in humans, present a compelling case for the idea that ZIKV infection is dependent on the genetic background. Further, the variable presentations of ZIKV infection observed in various animal models can be attributed to the individual and unique host immune responses as observed with other viral infections.

The *Ifnar1*^−/−^ mice show some promise as a test model, but the specificity of their mutation limits their capability to cases where vertical transmission is a concern (Miner et al., 2016). The other mouse strains that have been used to establish ZIKV infection are A129 and AG129 mice. The A129 mice fail to express the type I interferon receptor, while AG129 mice fail to express both type I and Type II interferon receptors. When infected with ZIKV, both of these models demonstrate death of young mice (3 weeks) and symptoms in older mice (>11 weeks) including viremia, weight loss, and illness (Rossi et al., 2016). However, these mouse models are not suitable for vaccine testing that relies on intact IFN pathway. IFN signaling is crucial to prime the innate and adaptive immune response that is required for the vaccine to exert its protective effect against infection.

Despite some shortcomings, the mouse models remain one of the most potent tools for understanding ZIKV infection and development of novel treatment modalities (LaMonica et al., 2012; Becker, 2016; Cugola et al., 2016; Lazear et al., 2016; Ramos da Silva and Gao, 2016; Rossi et al., 2016). The immunocompetent strains of mice that are susceptible to ZIKV infection and demonstrate clinical manifestations of ZIKV infection as observed in humans, including SJL mouse strains, appear very promising tools to gain deeper understanding of ZIKV infection.

Some researchers have suggested the use of chicken embryos as ZIKV infection model. Wild-type (WT) chicken embryos are shown to be susceptible to infection by ZIKV isolate MEX1-44, and the embryos display microcephaly-like outcomes (Goodfellow et al., 2016). The advantage of chicken embryos is that the neurodevelopment of chicken embryos is much more similar to the neurodevelopment of humans. Moreover, the gestational period is longer than in mice. The key issue is that there is no placenta for chicken embryos, and so they cannot be used for studies of the placental barrier. However, their homology to human neurodevelopment keeps them as a viable model for the understanding and prevention of neurodevelopmental issues arising from ZIKV (Goodfellow et al., 2016).

Researchers have attempted to develop brain organoids from pluripotent stem cells of chimpanzees (Cugola et al., 2016). The experiments showed promise in that the brain organoids displayed a slight reduction in the number of TBR1- and CTP2 cell percentages when the organoids were exposed to ZIKV. However, these findings were only seen when the organoids were infected with ZIKV^AF^. When the organoids were infected with a strain from the current epidemic (ZIKV^BR^), no infection was observed. This does not eliminate the possibility of a chimpanzee animal model, but the development of such a model would require modification of the virus or the progenitor cells. An interesting takeaway of this finding is that the ZIKV strains display vastly different virulence capabilities (Cugola et al., 2016).

The possibility of a Rhesus Macaque model has garnered a great deal of attention. Eight Rhesus Macaques were infected with the ZIKV strain H/PF/2013, including 2 pregnant subjects (https://zika.labkey.com). In the non-pregnant subjects, ZIKV was found to have disappeared within 21 days, and rechallenge failed to elicit infection in any of them. This finding is encouraging because it demonstrates protection from homologous reinfection. The two pregnant subjects were found to be positive for ZIKV for 57 days, with RNA persistence up to 71 days. Through ultrasound, it was determined that the fetuses had lower than normal biparietal diameters, head circumferences, and femur lengths. However, the difference from average was <3 standard deviations, so it cannot be said that the fetuses displayed microcephaly. The fetuses were free of ZIKV RNA starting at 10 days, and remained so with checks at 36 and 43 days. This is an interesting finding, because it presents the possibility that the placenta or the fetus may serve as a reservoir for ZIKV. This possibility is further supported by the biopsies, which demonstrate moderate levels of ZIKV in the placenta and decidua of one mother and both fetuses. Both mothers also displayed mild to moderate levels of ZIKV in the amniotic membrane and chorionic membrane.

The Rhesus Macaque model is currently showing a great deal of promise, both because of the susceptibility of the subjects to ZIKV and the homology of Rhesus Macaque neurodevelopment to human neurodevelopment. The major issue is that ZIKV virulence varies from host to host, so it remains to be determined if the same strains will demonstrate different effects in humans and Macaques. Additionally, Macaques are seasonal maters and require more habitat than mice, so the logistics of the Rhesus Macaque models may prove to be a barrier (Dudley et al., 2016; Ramos da Silva and Gao, 2016).

Recently a study established ZIKV infections in Dunkin-Hartley guinea pigs using inoculations of PRVABC59 strains of ZIKV subcutaneously (Kumar et al., 2017). Clinical signs such as fever, lethargy, ruffled fur, lack of mobility, and hunched back were evident in the guinea pigs. qRT-PCR and plaque assay of whole blood and serum found ZIKV and an anti-ZIKV neutralizing antibody was also identified with PRNT. The infections considerably raised the levels of several chemokines, growth factors, and cytokines found in the serum. The highest viral load was detected in the brain. Furthermore, ZIKV replication was also noted in the spleen. The conclusions found in this study no doubt demonstrate that guinea pigs are appropriate to further evaluate ZIKV pathogenesis.

## ZIKV proteins

A more robust understanding of the viral structure must be developed to address appropriately the current ZIKV outbreak as well as the outbreaks of ZIKV in the future. A key component of this is understanding the proteins that make up the ZIKV capsule. There are two major advantages that arise from better understanding of the structure of the ZIKV. First, a greater understanding of the component proteins allows for greater sensitivity and specificity in the detection and diagnosis of ZIKV infection. An important element of this is developing tests that will allow for differentiation between ZIKV and the other flaviviruses. Second, by understanding the structure and function of the component proteins of ZIKV, therapeutics can be developed that target important proteins. It is hoped that both of these advantages will allow us to minimize or eliminate the impact of ZIKV on humans.

There are currently 10 proteins of greatest interest for ZIKV. These 10 proteins can be classified into two groups: structural and non-structural proteins. The structural proteins comprise of an envelope protein, capsid protein, and precursor membrane protein. These proteins provide structure for the viral capsid. NS1, NS2A, NS2B, NS3, NS4A, NS4B, and NS5 are the non-structural proteins. They play a role in virulence, replication, secretion, or pathogenicity. The nonstructural proteins are the major factors of positive selection in ZIKV (Sironi et al., 2016). Of these proteins, NS1 and NS4A are the proteins of greatest interest as these proteins show marked mutation in the ZIKV^BR^ that is the cause of the current epidemic in Brazil.

The structure of NS1 is well conserved across flaviviruses, and IgM serology of NS1 is often used in the diagnosis of flavivirus infection. The size of NS1is generally between 46 and 55 kDa, and it may exist as an intracellular monomer, a membrane bound dimer, or a secreted hexamer. Three distinct regions are most conserved in NS1: a 1-29 amino acid β-roll, a 38-151 amino acid α/β Wing domain, and a 181-352 amino acid central β-ladder. NS1 is known to play a vital role in viral replication and virulence in the early stages of infection. In particular, glycosylated NS1 plays a role in RNA replication and modulation of host immune response (Akey et al., 2014). Several sites in ZIKV NS1 are unique from other flaviviruses, and it is postulated that these modifications to NS1 are positively selected (Sironi et al., 2016). They also found NS1 to be undesirable as a target for treatment because of the rapid rate of NS1 evolution (Sironi et al., 2016).

NS2B serves as a cofactor for the activation of NS3. NS3 contains viral protease and helicase function, both of which are vital for viral replication (Cox et al., 2015; Luo et al., 2015). The protease cleaves anchC-prm, NS2B-NS3, NS3-NS4A, NS4A-NS4B, and, NS4B-NS5 so the proteins may be used. The role of the NS3 protease is so vital that it is believed the virus will be incapable of replicating if protease inhibitors against NS3 are administered. For this reason, NS3 is a major target being studied for the treatment of ZIKV, as well as Dengue and West Nile virus. The mutations found in helicase portion of NS3 cause minimal structural difference, meaning NS3 structure is carefully conserved, and is likely not an ideal candidate for differentiating between the different flaviviruses.

NS4B is a transmembrane protein with 2 perimembrane domains, a cytosolic domain, and 3 transmembrane domains (Zmurko et al., 2015). In conjugation with p2K, it is responsible for anchoring of the replication complex, evasion of immune response, and various specialized interactions with host cell factors. The transmembrane elements, pTM1 and pTM2, have been noted to have an antagonistic effect on interferon (Cox et al., 2015). While inhibitors have been found for NS4B from other flaviviruses, those inhibitors have failed to work on ZIKV NS4B. Thus, it may be worthwhile to pursue the development of inhibitors for NS4B.

NS5 is a 903 residue protein with a methyltransferase on the N-terminal as well as a RNA dependent RNA polymerase domain (Cox et al., 2015). It is responsible for the modification of RNA and is essential for viral replication and evolution. The methyltransferase is vital for processing of viral RNA because of its GTP-transferase activity and RNA N-7 methyltransferase activity. The methyltransferase domain is a promising target because of the vital role it plays, but current inhibitors display poor penetration and high cross-reactivity (Brecher et al., 2015; Cox et al., 2015). The RNA-dependent RNA polymerase domain, vital for the formation of the RNA primer, has been established as a target for drug development, with current strategies utilizing allosteric and competitive inhibitors. This domain shows minimal variance from the RNA-dependent RNA polymerase domain of the Japanese encephalitis virus NS5 protein, meaning similar compounds should work for both viruses.

Precursor membrane (prM) protein is a 168 residue long structural protein that shares 42% homology between ZIKV and dengue virus. In ZIKV, a unique feature is present in prM protein. The first 93 residues serve to protect the protein from premature cleavage. When the intracellular pH drops in response to maturation, the protein is cleaved to allow the virus to mature completely. This unique feature of the prM protein in ZIKV may serve as a target for detection as well as treatment (Cox et al., 2015).

Env glycoprotein is a 504 residue long glycoprotein found as homodimers on the viral surface. Env glycoprotein plays a vital role in adhesion to the host cell, and is thus central to the virulence of ZIKV. The protein has three β-sheet domains including a centralized amino domain (domain I), a dimerization domain with a fusion sequence (domain II), and a transmembrane immunoglobulin-like domain (domain III) (Cox et al., 2015). Between Dengue virus and ZIKV, domain III displays differences in the exposed β-sheets, and thus, the cell-types to which each virus can adhere are altered. It is possible that mutations in this area are why the latest strain of ZIKV is capable of invading human cortical neural progenitor cells. A pocket between Domain I and Domain II that has been a popular target for treatment of Dengue virus is also altered in ZIKV to the point that drugs binding to the pocket do not work on ZIKV. While this does mean current treatments will not work, it also means chemical analogs that bind to this region may become target candidates to design effective therapeutic modalities for ZIKV infection (Cox et al., 2015). Recent developments have found that the domain EDIII of ZIKV envelope protein could be viable candidates for vaccines since these proteins are the central targets of neutralizing antibodies (Robbiani et al., 2017). Phase 1 trials for the DNA vaccine is currently underway (Tebas et al., 2017).

## Diagnostic tools

The diagnosis of ZIKV is part of what makes the management of the disease so difficult. Eighty percent of patients are asymptomatic, which means the majority of hosts do not even recognize that they are infected. In fact, many nations with widespread ZIKV have vastly underreported numbers (Fajardo et al., 2016). In symptomatic patients, clinical diagnosis is possible, but may be confounded in areas with dengue and chikungunya. In areas where it is possible, blood counts should be performed, though they will often do little to clarify the differential due to the similarity between the findings in many viral infections. Nonetheless, findings such as leukopenia, thrombocytopenia, hypoalbuminemia, and increase in transaminase levels and activated lymphocytes can serve as key markers for the presence of ZIKV. As the ZIKV evolves to develop greater virulence and morbidity, it becomes more important than ever to develop a reliable system of differential diagnosis to prevent outbreaks and vertical transmission. Moreover, the development of tests capable of detecting ZIKV infectivity can allow for greater sensitivity in research involving ZIKV (Fauci and Morens, 2016).

The most promising diagnostic tests are molecular approaches including RT-PCR and nucleic acid amplification test (NAT) (Table 4; Kurosaki et al., 2017; Liu et al., 2017; Ölschläger et al., 2017). Both of these methods display excellent sensitivity and specificity for ZIKV with minimal confounding by other flaviviruses (Liu et al., 2017). It has been demonstrated that the RT-PCR is capable of detecting viral RNA from 37 ZIKV strains (Faye et al., 2013). RT-PCR also avoided false positive that would have been caused by 19 other flaviviruses. The test is capable of detecting down to 337 pfu/mL and could detect viremia ranging from 10^3^ to 10^6^ pfu/mL (Faye et al., 2008). Logistically, the tests are inexpensive and may be completed with a variety of easily obtained biofluids, most notably, saliva.

**Table 4:** A summary of the various diagnostic tools available for the detection of ZIKV infection.

**Diagnostic tool**	**Confounding diagnoses**	**Sensitivity**	**Advantages**	**Disadvantages**	**References**
Clinical diagnosis	Many infections share the clinical symptoms of Zika, and most cases are asymptomatic	Low	Cheap, quick, effective during outbreaks	Difficult to clinically differentiate between Dengue or Chikungunya	Fauci and Morens, 2016; Musso and Gubler, 2016
Culture	None	Unknown	Cheap, highly specific	Often not useful in research. Culturing is difficult and not always successful	Musso and Gubler, 2016
RT-PCR	None	10^3^–10^6^ pfu/mL in natural human infection. 337 pfu/mL in assays	Cheap, fast, highly selective	Requires a laboratory, not all strains have been covered	Faye et al., 2008; Charrel et al., 2016
IgM and IgG ELISA	Other flaviviruses	High (titers must be greater than 20)	Fast, high sensitivity for flaviviruses	Difficult to clinically differentiate from other flaviviruses. Requires a laboratory	Duffy et al., 2009; Maeda and Maeda, 2013; Al-Qahtani et al., 2016
Plaque Reduction Neutralization Assay	Other flaviviruses	High (titers must be greater than 20)	Gold standard for flaviviruses. Widely used in research	Still under development. Expensive, slow, requires a specialized laboratory, not yet specialized for ZIKV	Duffy et al., 2009; Maeda and Maeda, 2013

Plaque-reduction neutralization tests (PRNT) and IgM ELISA are considered the gold standard for detection of flaviviruses (Maeda and Maeda, 2013; Joó et al., 2017). These assays have proven effective in a variety of outbreaks in the Pacific. In practice, titers greater than or equal to 20 of both IgM ZIKV ELISA and ZIKV PRNT are indicative of Zika if the ratio of ZIKV PRNT to Dengue virus PRNT is greater than or equal to 4 (https://www.cdc.gov/zika/laboratories/lab-guidance.html). These standards allow for effective differentiation between endemic flaviviruses and Zika. When the ratio is too low, however, the tests are usually abandoned (Duffy et al., 2009; Musso and Gubler, 2016). However, there are several key issues that make them less than ideal. Both of these tests often detect other flaviviruses in the place of ZIKV, resulting in misdiagnosis. Logistically, IgM ELISA and PRNT are difficult to accomplish due to the expense and paucity of the equipment. Both require specialized labs, PRNT more so than IgM ELISA. This barrier can be overcome in epidemics as dried blood samples on filter paper travel well and can be used in PRNT and IgM ELISA. However, the cost remains an important barrier for the implementation of these tests (Musso and Gubler, 2016). Ultimately, detection by PCR is the most useful for earliest diagnosis and IgM serology by ELISA is helpful soon after. Due to the increasing incidence of ZIKV infections, there is an urgent need to develop and improve diagnostic tools that can consistently spot ZIKV infection rapidly based on deep understanding about the pathogenesis of disease.

## Treatment

To date, no anti-ZIKV therapies are available. No vaccine has been approved thus far, and directed therapies to prevent and treat ZIKV infections are also deficient, posing an enormous threat to pregnant women and their developing infants. Management of clinical manifestations of ZIKV infections consists of only supportive care and there are no preventive measures that can be done against more severe and permanent consequences of ZIKV infections. The latest outbreak, therefore, prompts urgent development of a ZIKV vaccine. As such, the World Health Organization (WHO) has advocated the rapid development of ZIKV therapeutics (WHO, 2017). These include vaccines and other direct acting drugs that could to be studied in a systematic manner, and brought from bench to bedside. While it is a challenge to prevent adverse fetal outcomes due to this infection, studying pregnant mothers creates even a greater challenge, as most drugs/molecules available for potential ZIKA treatment are teratogenic, themselves. Furthermore, ZIKA infection in pregnancy is mostly asymptomatic with occasional fetal abnormalities with spontaneous clearance of the virus from blood within days and from urine within weeks. Having stated that, there are two modalities that have been considered as treatment and they include, but not limited to, to develop agents to prevent viral replication and the development of neutralizing antibodies against ZIKA to cause rapid neutralization of viruses.

Direct-acting antiviral are good contenders to be investigated for the treatment of ZIKA virus infection. Computational drug discovery has been extensively used to identify candidate drugs (Barrows et al., 2016; Ekins et al., 2016). In the absence of meaningful capital investment, repurposing drugs already approved, may be the most efficient method to design clinical trials and eventually bring them to bedside. Some of the drugs currently approved by the Food and Drug Administration (FDA) that are under consideration include, but not limited to, sofosbuvir (Sacramento et al., 2016), niclosamide (Xu et al., 2016), ivermectin, mefloquine, and daptomycin to name a few (Barrows et al., 2016; Table 5).

**Table 5:** Candidate Anti-ZIKV drugs and considerations for use in pregnancy.

**Drug name**	**Pregnancy category**	**Other considerations and notes**
Auranofin	C	Inform women of childbearing potential of the potential risk of therapy during pregnancy
Clofazimine	C	Some animal studies have failed to reveal evidence of teratogenicity, but studies done at high doses have demonstrated fetotoxicity. There is no controlled data in human pregnancy
Cyclosporine A	C	Advise of the potential risks if used during pregnancy
Daptomycin	B	
Deferasirox	C	
Digoxin	C	Concentrations with anti-ZIKV activity may be toxic
Fingolimod	C	A pregnancy registry has been established to collect information about the effect of this drug during pregnancy
Ivermectin	C	
Mebendazole	C	Inform of potential risk to fetus if taken during pregnancy, especially during first trimester
Mefloquine-HCI	B	
Methoxsalen	C	Usually given in combination with UV radiation therapy
Micafungin	C	
Palonosetron HCI	B	Drug interaction with SSRIs (Sertraline) causing serotonin syndrome
Pyrimethamine	C	Women of reproductive potential should avoid becoming pregnant while on therapy
Sertraline-HCI	C	Antidepressants increased the risk of suicidal thinking and behavior (suicidality) in children, adolescents, and young adults in short-term studies of major depressive disorder (DD) and other psychiatric disorders. Consider tapering does during third trimester of pregnancy

*FDA Pregnancy Categories: Category A-Adequate and well-controlled studies have failed to demonstrate a risk to the fetus in the first trimester of pregnancy (and there is no evidence of risk in later trimesters); Category B-Animal reproduction studies have failed to demonstrate a risk to the fetus and there are no adequate and well-controlled studies in pregnant women; Category C-Animal reproduction studies have shown an adverse effect on the fetus and there are no adequate and well-controlled studies in humans, but potential benefits may warrant use of the drug in pregnant women despite potential risks; Category D-There is positive evidence of human fetal risk based on adverse reaction data from investigational or marketing experience or studies in humans, but potential benefits may warrant use of the drug in pregnant women despite potential risks*.

When new agents are developed, it is worthwhile to remember the lessons learned from the HIV pandemic, where combination therapy was necessary to control viral replication due to rapid development of virus resistance. Another analogous and significant lesson was the subsequent expansion of these agents for pre-exposure prophylaxis, as a method of prevention. Perhaps this may be even a better strategy than trying to treat ZIKA virus infection in pregnancy, as it is much safer and could be more effective.

**Figure 4:**
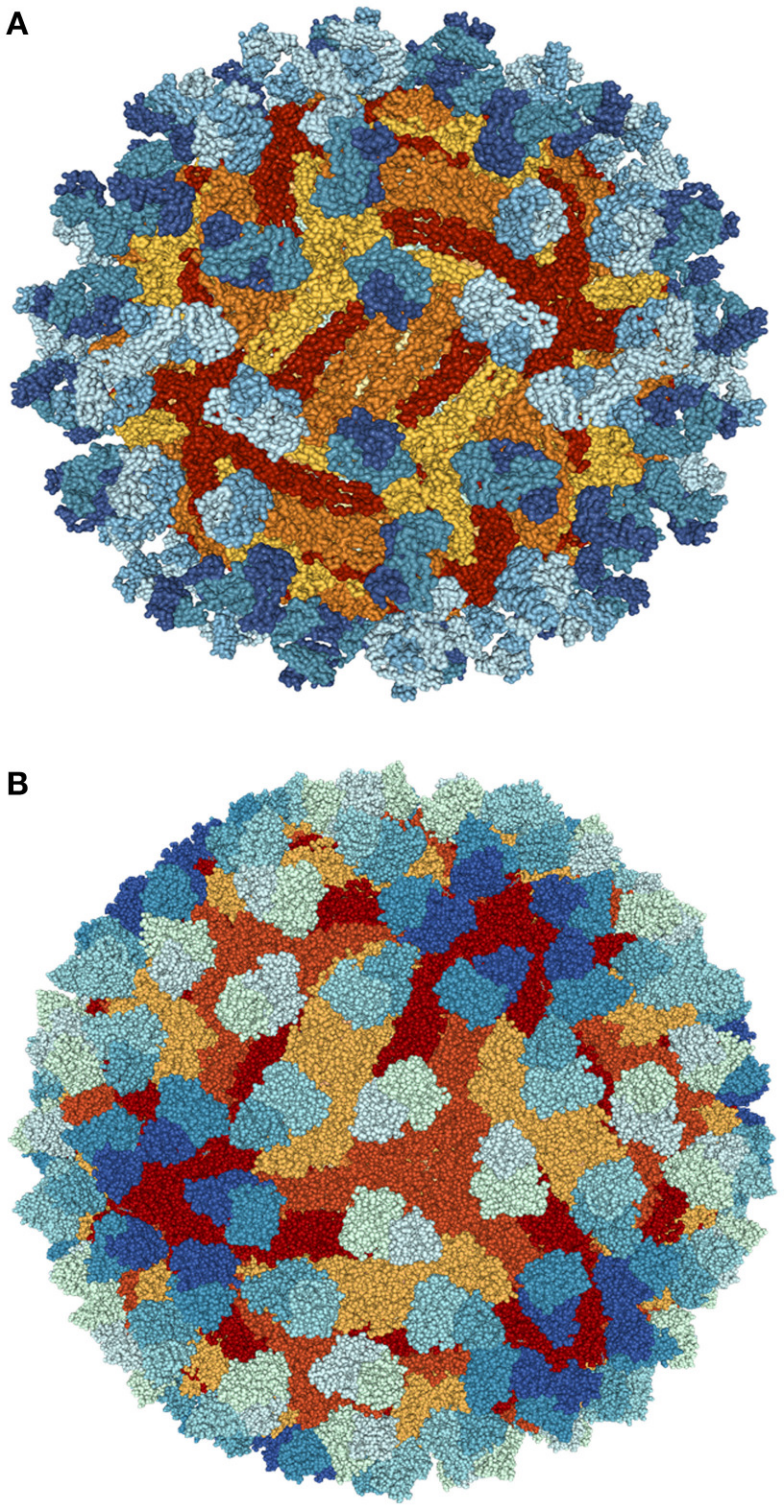
Structure of ZIKV complexed with antibody Fab C10 from Cryo-EM at pH 6.5 **(A)** and 8.0 **(B)** obtained from Protein data bank (PDB ids: 5H30 and 5H37). Blue shade (light to dark blue) shows antibody Fab C10; Red shade (yellow to red) highlights ZIKV-E (Envelope Protein E). Protein structures were visualized and rendered with NGL viewer (Rose and Hildebrand, 2015).

Recent encouraging research has found mice susceptible to ZIKV was recently found to have full protection against ZIKV complications via a single immunization using a plasmid DNA vaccine as well as a purified inactivated virus vaccine (Larocca et al., 2016). In order for a ZIKV vaccine for humans to be a viable option, however, further discernment of immune responses to ZIKV need to be established. Development of a live-attenuated vaccines have been stalled and paused lest there is further elucidation of the link between GBS and ZIKV. Additionally, complications related to pre-existing flavivirus exposure require deep probing in order to develop an efficient and safe vaccine (Martines et al., 2016). A highly potent Dengue virus human antibody C10 has been demonstrated to neutralize ZIKV efficiently (Figure 4; Barba-Spaeth et al., 2016; Swanstrom et al., 2016; Zhang et al., 2016). At pH 6.5, the antibody C10 is able to arrest all virus surface E proteins which prevents the E proteins from altering their structure during the most critical step of infection—the fusion event (Figure 4A). However, at pH 8.0, the ZIKV E proteins residues within the intra-dimer interface, the virus quaternary structure-dependent inter-dimer and inter-raft interfaces are bound to the C10 antibody (Figure 4B). However, additional studies are necessary to investigate the therapeutic potential of C10 antibody.

The main benefit of neutralizing antibodies against ZIKA is their relatively safe pharmacological profile, with the most concerning side effect being transfusion reactions, which are infrequent. In addition, neutralizing antibodies may be considered safe for the fetus (Faria et al., 2016), which further emphasizes them as a prospective therapeutic agent. Given their prior use in other maternal contagions, neutralizing antibodies may not need supplementary preclinical safety testing. The main restrictions of them include problems with collection, transportation and contamination with anti-dengue antibodies. A recent study demonstrated that pre-existing neutralizing antibodies (VH3-23/VK1-5) to dengue 1 virus (DENV1) correlated with high neutralizing responses to ZIKV in humans (Robbiani et al., 2017). The similarity in the lateral ridge region of the EDIII between ZIKV and DENV1 that is recognized by VH3-23/VK1-5 was suggested for this neutralization. Future investigations will help in determining the efficacy of neutralizing antibodies against ZIKV infection.

## Conclusions

With the swift occurrence of ZIKV infection as a progressing epidemic, the scientific world has been challenged with the need to concurrently study and understand a new disease, and to acquire effective treatment modalities. Many argue that Zika is a much more intricate challenge than Ebola, since it could influence more lives. ZIKV is vector-borne, and therefore its array of transmission will be limited by the vector ecosystem. To date, we have observed the most devastating effects of Zika-related disease on the unborn fetus. Unfortunately, the transplacental transmission of ZIKV is not well understood. Furthermore, ZIKV associated disease may have an autoimmune element, as indicated by the incidence of GBS. It is epidemic in a region with a high degree of global connectivity that can lead to widespread dissemination of ZIKV. The Zika epidemic is moving very rapidly. Unlike the recent Ebola epidemic, research reagents, animal models, and basic biological data are much less advanced against ZIKV. Zika virus has posed a great challenging situation not only for health and public sectors but also for economic sectors of different countries. The ZIKV outbreak has posed a significant burden on the healthcare system. The current Zika outbreak is the greatest of its kind, with 1.4 million cases in Brazil alone. There is a pressing need to understand the molecular mechanisms underlying ZIKV infection that will provide novel avenues for prevention and treatment leading to the protection of pregnant mothers and their developing infants.

## Author contributions

All authors listed have made a substantial, direct and intellectual contribution to the work, and approved it for publication.

### Conflict of interest statement

The authors declare that the research was conducted in the absence of any commercial or financial relationships that could be construed as a potential conflict of interest.
